# 3D polarization-interference holographic histology for wavelet-based differentiation of the polycrystalline component of biological tissues with different necrotic states. Forensic applications

**DOI:** 10.1117/1.JBO.29.5.052920

**Published:** 2024-03-15

**Authors:** Alexander Ushenko, Alexander Dubolazov, Jun Zheng, Alexandra Litvinenko, Mykhaylo Gorsky, Yuriy Ushenko, Iryna Soltys, Olexander Salega, Zhebo Chen, Oleh Wanchuliak

**Affiliations:** a Taizhou Institute of Zhejiang University, Taizhou, China; b Chernivtsi National University, Optics and Publishing Department, Chernivtsi, Ukraine; c Bucovinian State Medical University, Forensic Medicine and Medical Law Department, Chernivtsi, Ukraine; d Chernivtsi National University, Computer Science Department, Chernivtsi, Ukraine

**Keywords:** polarization, interference, holography, wavelet analysis, optical anisotropy, biological tissue, statistical moments, myocardium, lungs tissue

## Abstract

**Significance:**

The interference-holographic method of phase scanning of fields of scattered laser radiation is proposed. The effectiveness of this method for the selection of variously dispersed components is demonstrated. This method made it possible to obtain polarization maps of biological tissues at a high level of depolarized background. The scale-selective analysis of such maps was used to determine necrotic changes in the optically anisotropic architectonics of biological tissues.

**Objective:**

Development and experimental approbation of layered phase polarimetry of repeatedly scattered fields in diffuse layers of biological tissues. Application of scale-selective processing of the found coordinate distributions of polarization states in various phase sections of object fields. Determination of criteria (markers) for histological differential diagnosis of the causes of necrotic changes in optical anisotropy of biological tissues.

**Approach:**

We used a synthesis of three instrumental and analytical methods. Polarization-interference registration of laser radiation scattered by a sample of biological tissue. Digital holographic reconstruction and layered phase scanning of distributions of complex amplitudes of the object field. Analytical determination of polarization maps of various phase cross-sections of repeatedly scattered radiation. Application of wavelet analysis of the distributions of polarization states in the phase plane of a single scattered component of an object field. Determination of criteria (markers) for differential diagnosis of necrotic changes in biological tissues with different morphological structure. Two cases are considered. The first case is the myocardium of those who died as a result of coronary heart disease and acute coronary insufficiency. The second case is lung tissue samples of deceased with bronchial asthma and fibrosis.

**Results:**

A method of polarization-interference mapping of diffuse object fields of biological tissues has been developed and experimentally implemented. With the help of digital holographic reconstruction of the distributions of complex amplitudes, polarization maps in various phase sections of a diffuse object field are found. The wavelet analysis of azimuth and ellipticity distributions of polarization in the phase plane of a single scattered component of laser radiation is used. Scenarios for changing the amplitude of the wavelet coefficients for different scales of the scanning salt-like MHAT function are determined. Statistical moments of the first to fourth orders are determined for the distributions of the amplitudes of the wavelet coefficients of the azimuth maps and the ellipticity of polarization. As a result, diagnostic markers of necrotic changes in the myocardium and lung tissue were determined. The statistical criteria found are the basis for determining the accuracy of their differential diagnosis of various necrotic states of biological tissues.

**Conclusions:**

Necrotic changes caused by “coronary artery disease–acute coronary insufficiency” and “asthma–pulmonary fibrosis” were demonstrated by the method of wavelet differentiation with polarization interference with excellent accuracy.

## 1 Introduction

Polarization methods of diagnostics of optically anisotropic structure of biological tissues are widely used due to their high informativeness and sensitivity.^1^^–^^6^

Analysis of the polarization characteristics of scattered object fields allowed us to obtain quantitative criteria for assessing pathological changes in biological tissues. These methods have been used to successfully diagnose lens cataracts, quantify glucose concentrations in the biological tissues of diabetic patients, and identify malignant changes.^7^^–^^12^

A significant achievement in the field of biomedical diagnostics was the development of methods of Mueller image matrix microscopy (MMM).^13^^–^^17^ MMM includes a variety of constructive, physical, and spectral methods for measuring Mueller matrix images (MMI).^18^^–^^21^

Physical analysis of MMI is carried out within the framework of various theoretical models of the structure of biological tissues. The regression model of optical anisotropy,^6^ the model of an amorphous polycrystalline matrix,^5^^,^^22^^,^^23^ the logarithmic decomposition of the Mueller matrix,^15^^–^^17^ and the simulation of the transformation of polarized radiation by the Monte Carlo method^17^ can be distinguished among the main ones. For objective quantitative analysis of MMI, statistical^5^^,^^22^^–^^24^ correlation,^25^^–^^29^ Fourier^29^^–^^31^ singular,^5^^,^^22^ fractal,^5^^,^^23^ and wavelet analys^22^^,^^23^^,^^32^^–^^36^ are used.

The fundamental results obtained within the framework of MMM of biological tissues have wide diagnostic application in various fields of medicine.^37^ The possibility of obtaining quantitative optical indicators to characterize the evolution of gastric tissue from a healthy state through inflammation to cancer has been demonstrated.^14^ The criteria for the Mueller-matrix diagnosis of prostate cancer,^38^ bowel,^39^ and cervix^40^ have been determined. A method of differentiation of postmortem traumatic myocardial changes has been developed.^35^ Polarimetric criteria for determining the time of death have been found.^41^

The analysis of the literature data has shown that the further successful development of MMM is hindered by two main, as yet unresolved problems. The first problem is related to the high depolarizing ability of biological tissues.^1^^–^^4^^,^^11^^–^^16^^,^^42^^,^^43^ The presence of a high level of depolarized background reduces the depth of modulation (contrast) MMI.^43^^,^^44^ As a result, the sensitivity and accuracy of the polarization diagnosis of pathological conditions of biological tissues decreases.^44^^,^^45^ Studies in this area have revealed the dependence of the depolarization value on the parameters of the optical anisotropy of diffuse biological layers.^46^^,^^47^ To eliminate the effect of depolarization, a model of polar Mueller-matrix decomposition of the biological layer into various components: “polarizer – attenuator – depolarizer” was used here. This algorithmic approach made it possible to diagnose cancerous tissues.^48^ Another promising direction in eliminating the depolarized background may be the polarization-interference layer-by-layer phase selection of scattered laser fields. This approach is used to diagnose diffuse samples of benign and malignant prostate tumors.^49^

The second problem is that all the data of the image MMM are integrally averaged. The obtained quantitative criteria for changes in the structure of MMI are not directly related to different geometric scales of optically anisotropic architectonics of biological tissue.^6^^–^^17^^,^^25^^–^^31^^,^^50^^–^^52^ This circumstance limits the functionality of the MMM. A number of publications have shown that pathological changes in biological tissues manifest themselves differently at different scales of the polycrystalline structure.^5^^,^^22^^,^^23^^,^^32^^–^^36^ Scale-selective wavelet analysis^53^^–^^58^ of polarization maps and MMI of optically thin layers of biological preparations of tissues and liquids is used here. It is shown that the transformation of the amplitude of the wavelet coefficients of small-scale polycrystalline architectonics corresponds to early pathological changes in biological tissue.^32^^–^^34^ Polarization differentiation of formed tumors is more effective in analyzing transformations of the amplitudes of wavelet coefficients for large-scale structures of biological crystals.^35^^,^^36^

Thus, for the further development of the imitative MMM of diffuse biological tissues, it is relevant to combine the considered “depolarization” and “wavelet” methods into a single polarimetric technology.

In our work, we propose a new method for implementing this task. Our method is based on the principles of phase scanning of repeatedly scattered object fields of biological tissues. For this purpose, polarization-interference registration of the laser object field is carried out. According to the obtained interferograms, the digital holographic method reconstructs the distributions of complex amplitudes of such a field. The phase scanning of such a field ensures the selection of variously scattered components. In the phase plane of single scattering, the polarization maps of the optically anisotropic architectonics of biological tissue are algorithmically determined. For the found azimuth and ellipticity distributions of polarization, the wavelet decomposition algorithm is used. A salt-like MHAT function with a variable scale (b) of the coordinate scanning window (a) is used as a wavelet.^53^^–^^58^ At each scale (b) of the MHAT function, the linear dependences (a) of the amplitudes of the wavelet coefficients of the azimuth maps and the ellipticity of the polarization are determined. Based on the obtained dependencies, the central statistical moments of the first and second orders are calculated, which characterize the mean and variance of fluctuations of different-scale amplitudes of the wavelet coefficients. Thus, the most sensitive diagnostic markers of structural changes in the optically anisotropic architectonics of diffuse biological tissue are determined. Further, within the framework of evidence-based medicine algorithms, the operational characteristics of the diagnostic power of the method are calculated.

Our work is aimed at the fundamental development and experimental approbation of this method of polarization-interference polarimetry of repeatedly scattered fields. As objects, we considered optically thick depolarizing samples of native histological sections of myocardium and lung tissue with different optically anisotropy architectonics.

The applied aspect of the work consisted of determination of criteria (markers) for differential diagnosis of necrotic changes in biological tissues with different morphological structure. Two cases are considered. The first case is the myocardium of those who died as a result of coronary heart disease (CHD) and acute coronary insufficiency (ACI). The second case is lung tissue samples of deceased with bronchial asthma (BA) and fibrosis.

## 2 Materials and Methods

### 2.1 Brief Theory

We briefly consider (without reducing the completeness of the analysis) within the framework of the linear birefringence LB approximation,^6^^,^^19^^–^^25^^,^^50^^–^^52^ the main theoretical provisions of our work.

#### 2.1.1 Stokes polarimetry of the object field

We chose a right-circularly (⊗) polarized laser beam as the radiation illuminating biological tissues. This condition is necessary when measuring a series of samples of biological tissues. For other states of polarization, the result of its object transformation will be azimuthally dependent on the rotation of the sample relative to the direction of irradiation.^44^^,^^45^

The Stokes vector of such beam has the following form:^4^^,^^5^^,^^7^^–^^12^

$$S^{0}(⊗)=(\begin{array}{c} 1\\ 0\\ 0\\ 1 \end{array}).$$ (1)

#### 2.1.2 Single scattering

For the case of single scattering (j=1), the polarization properties of the linear birefringence protein fibrilla in the point with coordinate (r) correspond to the Mueller matrix operator^6^^,^^19^^–^^25^^,^^50^^–^^52^ $(\{W\})(r{)}^{j=1}$:

$$\left(\left\{W\right\}\right){\left(r\right)}^{j=1}={\left(\left[\begin{array}{cccc} 1 & 0 & 0 & 0\\ 0 & \omega_{22} & \omega_{23} & \omega_{24}\\ 0 & \omega_{32} & \omega_{33} & \omega_{34}\\ 0 & \omega_{42} & \omega_{43} & \omega_{44} \end{array}\right]\right)}^{j=1}\;(r)={\left([\begin{array}{cccc} 1 & 0 & 0 & 0\\ 0 & \left(\cos^{2}\;2\rho +\sin^{2}\;2\rho \;\cos\;\delta \right) & \left(\cos\;2\rho \;\sin\;2\rho (1-\cos\;\delta )\right) & \left(\sin\;2\rho \;\sin\;\delta \right)\\ 0 & \left(\cos\;2\rho \;\sin\;2\rho (1-\cos\;\delta )\right) & \left(\sin^{2}\;2\rho +\cos^{2}\;2\rho \;\cos\;\delta \right) & \left(\cos\;2\rho \;\sin\;\delta \right)\\ 0 & \left(\sin\;2\rho \;\sin\;\delta \right) & \left(\cos\;2\rho \;\sin\;\delta \right) & \left(\cos\;\delta \right) \end{array}]\right)}^{j=1}(r)$$ (2)

Here, $\omega_{lk}$ is the elements of the Mueller matrix with column (l=1;2;3;4) and row (k=1;2;3;4) indexes; ρ is the orientation of the optical axis of the birefringent fibril; $\delta =\frac{2\pi }{\lambda }\Delta nd$ is the phase shift; Δn is the linear birefringence index LB; d is the geometrical size; and λ is the wavelength.

The process of single transformation in local point (r) of the probing beam $S^{0}(⊗)$ is described by the following matrix equation:

$$(S^{*}(r){)}^{j=1}=\{W\}(r{)}^{j=1}S^{0}(⊗).$$ (3)

Here, $(S^{*}(r){)}^{j=1}$ is the Stokes vector of a single scattered component of an object field at a point (r):

$$(S^{*}(r){)}^{j=1}={\left((\begin{array}{c} S_{1}^{*}\\ S_{2}^{*}\\ S_{3}^{*}\\ S_{4}^{*} \end{array})(r)\right)}^{j=1}={\left(S_{1}^{*}\right)}^{-1}{\left((\begin{array}{c} 1\\ \cos\;2\alpha \;\cos\;2\beta \\ \sin\;2\alpha \;\cos\;2\beta \\ \sin\;2\beta \end{array})\right)}^{j=1}(r),$$ (4)

where α(r) is the azimuth and β(r) is the ellipticity of polarization.

In the expanded form Eq. (3), taking into account Eqs. (2) and (4), can be rewritten as follows:

$$(\begin{array}{c} S_{1}^{*}\\ S_{2}^{*}\\ S_{3}^{*}\\ S_{4}^{*} \end{array})(r)=(\begin{array}{c} 1\\ \omega_{24}\\ \omega_{34}\\ \omega_{44} \end{array})(r)=(\begin{array}{c} 1\\ \left(\sin\;2\rho \;\sin\;\delta \right)\\ \left(\cos\;2\rho \;\sin\;\delta \right)\\ \cos\;\delta ) \end{array})(r).$$ (5)

As a result, we find the relationship between the polarization parameters ($\alpha^{j=1}$, $\beta^{j=1}$) and the characteristics of linear birefringence (ρ,δ) at the point (r) in the form of the following analytical relations:

$$\alpha^{j=1}(r,\rho )=0.5\;\arctan((S_{3}^{*}(r)/S_{2}^{*}(r)){)}^{j=1}=0.5\;\arctan(\mathrm{cotan}2\rho (r)),$$ (6)

$$\beta^{j=1}(r,\delta )=0.5\;\arcsin((S_{4}^{*}(r)/S_{1}^{*}(r)){)}^{j=1}=0.5\;\arcsin(\cos\;\delta (r)).$$ (7)

The distributions of the polarization parameters $\alpha^{j=1}(r,\rho )$ and $\beta^{j=1}(r,\delta )$ at all points r∈R of the once scattered component of the object field can be written in symbolic form $A^{j=1}(\alpha^{j=1}(R,\rho ))$ and $B^{j=1}(\beta^{j=1}(R,\delta ))$.

Thus, a polarization-structural map of an optically thin layer of birefringent architectonics of biological tissue is formed:

$$P^{j=1}(R,\alpha^{j=1},\beta^{j=1})\Leftrightarrow (\begin{array}{c} A^{j=1}(\alpha^{j=1}(R,\rho ))\\ B^{j=1}(\beta^{j=1}(R,\delta )) \end{array}).$$ (8)

#### 2.1.3 Multiple scattering

For series multiple acts (j≥1;2;3,…p−1,p) of interaction with the fibrillar network of a circularly polarized laser probe $S^{0}(⊗)$, the matrix Eq. (3) takes the form^6^

$$(\begin{array}{c} S^{j=1}(r,\alpha^{j=1};\beta^{j=1})\\ S^{j=2}(r,\alpha^{j=2};\beta^{j=2})\\ \vdots \\ S^{j=p-1}(r,\alpha^{j=p-1};\beta^{j=p-1})\\ S^{j=p}(r,\alpha^{j=p};\beta^{j=p}) \end{array})=(\begin{array}{c} \{W{\}}^{j=1}\\ \{W{\}}^{j=2}\{W{\}}^{j=1}\\ \vdots \\ \{W{\}}^{j=p-1}\ldots \{W{\}}^{j=2}\{W{\}}^{j=1}\\ \{W{\}}^{j=p}\{W{\}}^{j=p-1}\ldots \{W{\}}^{j=2}\{W{\}}^{j=1} \end{array})(r)S^{0}(⊗).$$ (9)

Here, $S^{j}(r,\alpha^{j};\beta^{j})$ is the set of Stokes vectors and polarization parameters at the point r. $\{W{\}}^{j}$ is a partial Muller matrix operator of the form Eq. (3) for each j’th scattering act.

Thus, at each point r repeatedly (j≥1;2;3,…p−1,p) of the scattered field, the azimuth and ellipticity of polarization are averaged to certain values $\alpha^{p}(r)$ and $\beta^{p}(r)$:

$$\alpha^{j=p}(r,\rho )=0.5\;\arctan(\sum_{j=1}^{p}(S_{3}^{*}{)}^{j}(r)/\sum_{j=1}^{p}(S_{2}^{*}{)}^{j}(r));$$ (10)

$$\beta^{j=p}(r,\delta )=0.5\;\arcsin(\sum_{j=1}^{p}(S_{4}^{*}{)}^{j}(r)/\sum_{j=1}^{p}(S_{1}^{*}{)}^{j}(r)).$$ (11)

As a result, a polarization-inhomogeneous component of the diffuse field will be formed with a different distribution of azimuth values $A^{j=p}(\alpha^{j=p}(R,\rho ))$ and ellipticity $B^{j=p}(\beta^{j=p}(R,\delta ))$ polarization.

Thus, a polarization-structural map of the diffuse layer of birefringent biological tissue is formed

$$P^{j=p}(R,\alpha^{j=p},\beta^{j=p})\Leftrightarrow (\begin{array}{c} A^{j=p}(\alpha^{j=p}(R,\rho ))\\ B^{j=p}(\beta^{j=p}(R,\delta )) \end{array}).$$ (12)

The conducted consideration [ratios (1)–(12)] describes the process of direct formation by optically anisotropic architectonics of the “object component” P(R,α,β) of a polarization inhomogeneous field

$$P(R,\alpha ,\beta )=(\begin{array}{c} A^{j=1}(\alpha^{j=1}(R,\rho ))\\ B^{j=1}(\beta^{j=1}(R,\delta )) \end{array})+(\begin{array}{c} A^{j=p}(\alpha^{j=p}(R,\rho ))\\ B^{j=p}(\beta^{j=p}(R,\delta )) \end{array}).$$ (13)

In parallel with the direct acts of interaction of laser radiation with birefringent biological crystals [ratios (1)–(12)], secondary interference of coherent scattered waves occurs.

The result of this process is the amplitude addition of variously polarized (ratio (13)) partial coherent waves.

#### 2.1.4 Amplitude consideration

For coherent laser fields, there is a direct relationship between the values of the Stokes vector parameters and the orthogonal components ($U_{x}^{j=1}$ and $U_{y}^{j=1}$) of complex amplitudes.^27^^–^^31^ Based on this, the expressions (6) and (7) obtained earlier for the polarization parameters can be rewritten in the following form:

$$\alpha^{j=1}(r,\rho )=0.5\;\arctan(((U_{x}^{j=1})(U_{y}^{j=1}{)}^{*}+(U_{x}^{j=1}{)}^{*}(U_{y}^{j=1}))/((U_{x}^{j=1})(U_{x}^{j=1}{)}^{*}-(U_{y}^{j=1})(U_{y}^{j=1}{)}^{*})).$$ (14)

$$\beta^{j=1}(r,\rho )=0.5\;\arcsin(i((U_{x}^{j=1})(U_{y}^{j=1}{)}^{*}-(U_{x}^{j=1}{)}^{*}(U_{y}^{j=1}))/((U_{x}^{j=1})(U_{x}^{j=1}{)}^{*}+(U_{y}^{j=1})(U_{y}^{j=1}{)}^{*})).$$ (15)

Here, i is an imaginary unit and * is a complex conjugate quantity.

#### 2.1.5 Interference interaction

For the orthogonal amplitude components $U_{x}^{j=1}$ and $U_{y}^{j=1}$, the following interference equations can be written:

$$U_{x}^{j=1}=(U_{x1}^{j=1}+U_{x2}^{j=1})=(|U_{x1}^{j=1}|+|U_{x2}^{j=1}|+2\sqrt{\left|U_{x1}^{j=1}||U_{x2}^{j=1}\right|}\cos\;\delta_{x12});$$ (16)

$$U_{y}^{j=1}=(U_{y1}^{j=1}+U_{y2}^{j=1})=(|U_{y1}^{j=1}|+|U_{y2}^{j=1}|+2\sqrt{\left|U_{y1}^{j=1}||U_{y2}^{j=1}\right|}\cos\;\delta_{y12}),$$ (17)

where $\left|U_{x1}^{j=1}|;|U_{y1}^{j=1}\right|$ are the modules of complex amplitudes; $\delta_{x12}$ и $\delta_{y12}$ are the values of phase shifts between $\left(U_{x1}^{j=1};U_{x2}^{j=1}\right)$ and $\left(U_{y1}^{j=1};U_{y2}^{j=1}\right)$.

For the process of forming the resulting values of the orthogonal components of the amplitudes $U_{x}$ and $U_{y}$ as a result of the j=p interaction, we can write

$$U_{x}^{j=p}=\overset{p}{\underset{j=1}{\sum }}U_{x}^{j};\;U_{y}^{j=p}=\overset{p}{\underset{j=1}{\sum }}U_{y}^{j};\;\delta_{xy}^{p}=\overset{p}{\underset{j=1}{\sum }}\delta_{x}^{j}-\overset{p}{\underset{j=1}{\sum }}\delta_{y}^{j}.$$ (18)

The interference addition of two phase-shifted $\varphi_{xy}^{p}$ orthogonal components $U_{x}^{j=p}$ and $U_{y}^{j=p}$ forms an elliptically polarized wave^6^

$$(X^{2}/(U_{x}^{j=p}{)}^{2})+(Y^{2}/(U_{y}^{j=p}{)}^{2})-2(XY/U_{x}^{j=p}U_{y}^{j=p})\cos\;\delta_{xy}^{p}=\sin^{2}\;\delta_{xy}^{p},$$ (19)

with next interference means (I) of azimuth $\alpha^{j=p}(I)$ and ellipticity $\beta^{j=p}(I)$

$$\alpha^{j=p}(I)=0.5\;\arcsin(\sin\;2(U_{y}^{j=p}/U_{x}^{j=p})/\sqrt{1+\tan^{2}\;\delta_{xy}^{p}\;\cos^{2}2(U_{y}^{j=p}/U_{x}^{j=p})}),$$ (20)

$$\beta^{j=p}(I)=0.5\;\arctan(\tan\;\delta_{xy}^{p}\;\sin\;2(U_{y}^{j=p}/U_{x}^{j=p})/\sqrt{1+\tan^{2}\;\delta_{xy}^{p}\;\cos^{2}\;2(U_{y}^{j=p}/U_{x}^{j=p})}).$$ (21)

Thus, a secondary polarization-inhomogeneous component [ratios (20) and (21)] of a diffuse object field with probabilistic distributions of azimuth and ellipticity values is interferentially formed

$$P(I{)}^{j=p}(R,\alpha (I{)}^{j=p},\beta (I{)}^{j=p})\Leftrightarrow (\begin{array}{c} A(I{)}^{j=p}(\alpha (I{)}^{j=p}(R,\rho ))\\ B(I{)}^{j=p}(\beta (I{)}^{j=p}(R,\delta )) \end{array}).$$ (22)

#### 2.1.6 Resulting field

So, the polarization structure of the laser field diffusely scattered by the tissue layer can be represented as a superposition of the following components:

$$\Phi (R,\alpha ;\beta )=(\begin{array}{c} A^{j=1}(\alpha^{j=1}(R,\rho ))\\ B^{j=1}(\beta^{j=1}(R,\delta )) \end{array})+(\begin{array}{c} A^{j=p}(\alpha^{j=p}(R,\rho ))\\ B^{j=p}(\beta^{j=p}(R,\delta )) \end{array})+(\begin{array}{c} A(I{)}^{j=p}(\alpha (I{)}^{j=p}(R,\rho ))\\ B(I{)}^{j=p}(\beta (I{)}^{j=p}(R,\delta )) \end{array}).$$ (23)

#### 2.1.7 Wavelet analysis of polarizing maps

The wavelet decomposition provides the mathematical possibility of large-scale selective analysis of a polarization-inhomogeneous field [relation (23)]. A salt-like MHAT function with a variable scale (b) of the coordinate scanning window (a) is used as a wavelet. At each scale (b) of the MHAT function (Ψ), the linear dependences (a) of the amplitudes of the wavelet coefficients of the azimuth maps and the ellipticity of the polarization are determined.^32^^–^^36^

The continuous wavelet transforms of the function Φ(α;β) is defined by the following equation:^53^^–^^58^

$$W(a,b)=\frac{1}{\sqrt{a}}\int_{-\infty }^{\infty }\Phi (x)\Psi (\frac{x-b}{a})dx,$$ (24)

where a is a scale parameter, b is a spatial coordinate, and Ψ is a soliton-like function (wavelet) constructed on the basis of derivatives of the Gaussian function.

In our work, the second derivative (z=2) or MHAT wavelet is used

$$\Psi^{\left(z\right)}=(-1{)}^{z}\frac{\partial^{z}}{\partial x^{z}}[\exp(\frac{x^{2}}{2})]\Rightarrow \Psi^{\left(2\right)}=\frac{\partial^{2}}{\partial x^{2}}[\exp(\frac{x^{2}}{2})].$$ (25)

The wavelet relations (24) and (25) for polarization maps of azimuth and ellipticity [ratio (23)] can be written as the following expressions:

$$\left\{ \begin{array}{c} W^{\alpha }(a,b)=\frac{1}{\sqrt{a}}\int_{-\infty }^{\infty }(A^{j=1}(\alpha^{j=1},x)+A^{j=p}(\alpha^{j=p},x)+A(I{)}^{j=p}(\alpha (I{)}^{j=p},x)+)\Psi (\frac{x-b}{a})dx;\\ W^{\beta }(a,b)=\frac{1}{\sqrt{a}}\int_{-\infty }^{\infty }(B^{j=1}(\beta^{j=1},x)+B^{j=p}(\beta^{j=p},x)+B(I{)}^{j=p}(\beta (I{)}^{j=p},x)+)\Psi (\frac{x-b}{a})dx. \end{array}\right.$$ (26)

The analysis of expression (26) shows that the wavelet analysis of polarization maps is a superposition of a large-scale selective evaluation of the components of the object field with different light scattering multiplicity. In the following, we focus on the search for the possibilities of optical separation of the components of the object field with a low scattering multiplicity or scattered once.

### 2.2 Experimental Setup and Measurement Methodology

The methodology for polarization-interference measurement of the distributions (m,n-CCD pixels quantity) of the Stokes vector parameters [polarization maps α(m,n) and β(m,n)] is presented in Refs. 27, 28, 35, 36, 38, 44, 45, 49. However, detailed information is not provided in this work. For a better understanding of the further discussion, we provide a brief overview of the three-dimensional (3D) digital holographic scanning method.

**Fig. 1:**
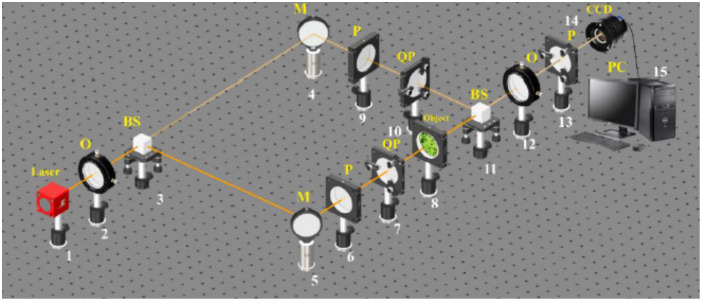
Optical scheme for polarization-interference mapping of the Stokes vector parameters. (1) He–Ne laser; (2) collimator – “O”; (3), (11) beam splitters “BS”; (4), (5) mirrors – “M”; (6), (9), (13) polarizers “P”; (7), (10) quarter wave plates – “QP”; (8) object; (12) polarization objective – “O”; (14) digital camera – “CCD”; (15) personal computer – “PC.”

A generalization of the polarization interferometry scheme^27^^,^^28^ is the Stokes-polarimetric mapping scheme on the base of Mach–Zehnder interferometer, which is shown in Fig. 1.

Parallel ($Ø=2\times 10^{3}\;\mu m$) beam of He–Ne (λ=0.6328  μm) laser 1, formed by spatial-frequency filter 2, with 50% beam splitter 3 is divided into “object” and “reference” ones.

The “object” beam with the help of a rotating mirror 5 is directed through the polarizing filter 6 - 7 (manufacturer: Achromatic True Zero-Order Waveplate and manufacturer: B + W Kaesemann XS-Pro Polarizer MRC Nano) in the direction of the biological layer 8 sample. The polarization-inhomogeneous image of biological tissue histological Sec. 8 is projected by the strain-free objective 12 (manufacturer: Nikon CFI Achromat P, focal length: 30 mm, numerical aperture: 0.1, magnification: 4×) into the digital camera 14 [The Imaging Source DMK 41AU02. AS, monochrome 1/2 “CCD, Sony ICX205AL (progressive scan) resolution: 1280×960; size of the photosensitive area: 7600×6200  μm; sensitivity: 0.05 lx; dynamic range: 8 bit, SNR: 9 bit); the photosensitive area of which contains m×n=1280×960  pixels) plane.

The “reference” beam is directed by the mirror 4 through the polarization filter 9–10 (manufacturer: Achromatic True Zero-Order Waveplate and manufacturer: B + W Kaesemann XS-Pro Polarizer MRC Nano) into the polarization image plane of biological tissue histological section 8.

As a result, an interference pattern is formed, the coordinate intensity distribution of which is recorded by a digital camera 14 through a polarizer 13.

Before carrying out measurements of biological tissues, the experimental device passed metrological certification with the introduction of model objects (“clean air,” “linear polarizer,” “phase plates 0.25λ,” “0.5λ”). As 50 measurements result for each type of object, the polarization ellipticity errors were determined β=0.0003  rad.

### 2.3 Method of Object Field 3D Polarimetry Phase Scanning

1. Using by polarizing filters 6–7 and 9–10, the circular polarization (⊗) is sequentially formed in the “irradiating” (Ir) and “reference” (Re) parallel laser beams—Ir(⊗)−Re(⊗).
2. For the circular polarization (⊗) state, two partial interference patterns are recorded through the polarizer-analyzer 14 with the orientation of the transmission plane at angles Ω=0  deg; Ω=90  deg.
3. Analytical processing of interference patterns was carried out using the digital Fourier transform FT(υ,ν):^27^^,^^28^^,^^35^^,^^36^^,^^38^^,^^44^^,^^45^^,^^49^
  $${\mathrm{FT}}_{x;y}(\upsilon ,\nu )=\frac{1}{M\times N}\overset{M-1}{\underset{m=0}{\sum }}\overset{N-1}{\underset{n=0}{\sum }}I_{x,y;\Omega =0\;\deg;90\;\deg}(m,n)\exp[-i2\pi (\frac{m\times \upsilon }{M}+\frac{n\times \nu }{N})],$$ (27)
  where $\left\{ \begin{array}{l} I_{x,\Omega =0\;\deg(m,n)}^{⊗}=(U_{x,\Omega =0\;\deg}^{⊗}){\left(U_{x,\Omega =0\;\deg}^{⊗}\right)}^{*};\\ I_{y,\Omega =90\;\deg(m,n)}^{⊗}=(U_{y,\Omega =90\;\deg}^{⊗}){\left(U_{y,\Omega =90\;\deg}^{⊗}\right)}^{*}; \end{array}\;U_{x,y;\Omega =0\;\deg;90\;\deg}^{⊗}\right.$ is the orthogonal components of complex amplitude; * denotes the complex conjugation operation; and (υ,ν) are the spatial frequencies.
4. The results of the digital Fourier transform (relation (27)) are used to obtain complex amplitudes distributions according to the following algorithms:
  $$U_{0\;\deg}(m,n)\to |U_{x,\Omega =0\deg}^{⊗}|(m,n);$$ (28)
  $$U_{90\;\deg}(m,n)\to (|U_{y,\Omega =90\;\deg}^{⊗}|\exp(i(\delta_{x}^{⊗}-\delta_{y}^{⊗})))(m,n).$$ (29)
5. By means of stepwise (Δδ) phase ($\delta_{t}$) scanning of the complex amplitudes [relations (28) and (29)] reconstructed field using algorithms (10) and (11), we obtain the coordinate distributions of polarization parameters $\alpha (\delta_{t},m,n)$ and $\beta (\delta_{t},m,n)$.
6. The resulting set of polarization maps $\Phi \equiv \{\begin{array}{c} \alpha (\delta_{t},m,n);\\ \beta (\delta_{t},m,n) \end{array}$ was analyzed in a statistical approach using the following algorithms to calculate mean ($Z_{1}$), variance ($Z_{2}$), skewness ($Z_{3}$), and kurtosis ($Z_{4}$.):^6^
  $$Z_{1}=\{\begin{array}{c} \frac{1}{m\times n}\overset{m\times n}{\underset{h=1}{\sum }}(\alpha (\delta_{t}){)}_{h};\\ \frac{1}{m\times n}\overset{m\times n}{\underset{h=1}{\sum }}(\beta (\delta_{t}){)}_{h}; \end{array}\;Z_{2}=\{\begin{array}{c} \sqrt{\frac{1}{m\times n}\overset{m\times n}{\underset{h=1}{\sum }}((\alpha (\delta_{t}){)}_{h}{)}^{2};}\\ \sqrt{\frac{1}{m\times n}\overset{m\times n}{\underset{h=1}{\sum }}((\beta (\delta_{t}){)}_{h}{)}^{2};} \end{array}\;Z_{3}=\{\begin{array}{c} \frac{1}{Z_{2}^{3}}\frac{1}{m\times n}\overset{m\times n}{\underset{h=1}{\sum }}((\alpha (\delta_{t}){)}_{h}{)}^{3};\\ \frac{1}{Z_{2}^{3}}\frac{1}{m\times n}\overset{m\times n}{\underset{h=1}{\sum }}((\beta (\delta_{t}){)}_{h}{)}^{3}; \end{array}\;Z_{4}=\{\begin{array}{c} \frac{1}{Z_{2}^{4}}\frac{1}{m\times n}\overset{m\times n}{\underset{h=1}{\sum }}((\alpha (\delta_{t}){)}_{h}{)}^{4};\\ \frac{1}{Z_{2}^{4}}\frac{1}{m\times n}\overset{m\times n}{\underset{h=1}{\sum }}((\beta (\delta_{t}){)}_{h}{)}^{4}. \end{array}$$ (30)
7. The object field is scanned to such a value of the phase shift $\delta_{t}^{⋆}$, starting from which the statistical condition of a single scattering in the volume of biological tissue is realized:
  $$Z_{i=1;2;3;4}(\alpha (\delta_{t}\leq \delta_{t}^{⋆}))\approx \text{const}\;\text{ and }\;Z_{i=1;2;3;4}(\beta (\delta_{t}\leq \delta_{t}^{⋆}))\approx \mathrm{const}.$$ (31)
8. Implement the wavelet transform of polarization maps [ratio (23)]
  $$W^{\alpha }(a,b,\delta_{t}^{⋆})\Rightarrow \frac{1}{\sqrt{a}}\int_{-\infty }^{\infty }A(\alpha ,a,x)\Psi (\frac{x-b}{a})dx;$$ (32)
  $$W^{\beta }(a,b,\delta_{t}^{⋆})\Rightarrow \frac{1}{\sqrt{a}}\int_{-\infty }^{\infty }B(\beta ,a,x)\Psi (\frac{x-b}{a})dx.$$ (33)
9. For various a MHAT scales, the mean $Z_{1}$ and variance $Z_{2}$ of coordinate dependencies are calculated:
  $$Z_{1}=\frac{1}{m\times n}\overset{m\times n}{\underset{h=1}{\sum }}W^{\alpha }(a,b,\delta_{t}^{⋆}{)}_{h};\;Z_{1}=\frac{1}{m\times n}\overset{m\times n}{\underset{h=1}{\sum }}W^{\beta }(a,b,\delta_{t}^{⋆}{)}_{h},$$ (34)
  $$Z_{2}=\sqrt{\frac{1}{m\times n}\overset{m\times n}{\underset{h=1}{\sum }}(W^{\alpha }(a,b,\delta_{t}^{⋆}{)}^{2}{)}_{h};}\;Z_{2}=\sqrt{\frac{1}{m\times n}\overset{m\times n}{\underset{h=1}{\sum }}(W^{\beta }(a,b,\delta_{t}^{⋆}{)}^{2}{)}_{h}.}$$ (35)

### 2.4 Objects of Investigations

To implement the complex study of the myocardium histological sections samples:

• CHD: group 1, 12 samples;
• ACI: group 2, 12 samples.

The second two groups consisted of lung tissue histological sections of those who died from myocardial infarction and with the following concomitant pathologies:

• BA: group 3, 15 samples;
• pulmonary fibrosis (PF): group 4, 15 samples.

**Fig. 2:**
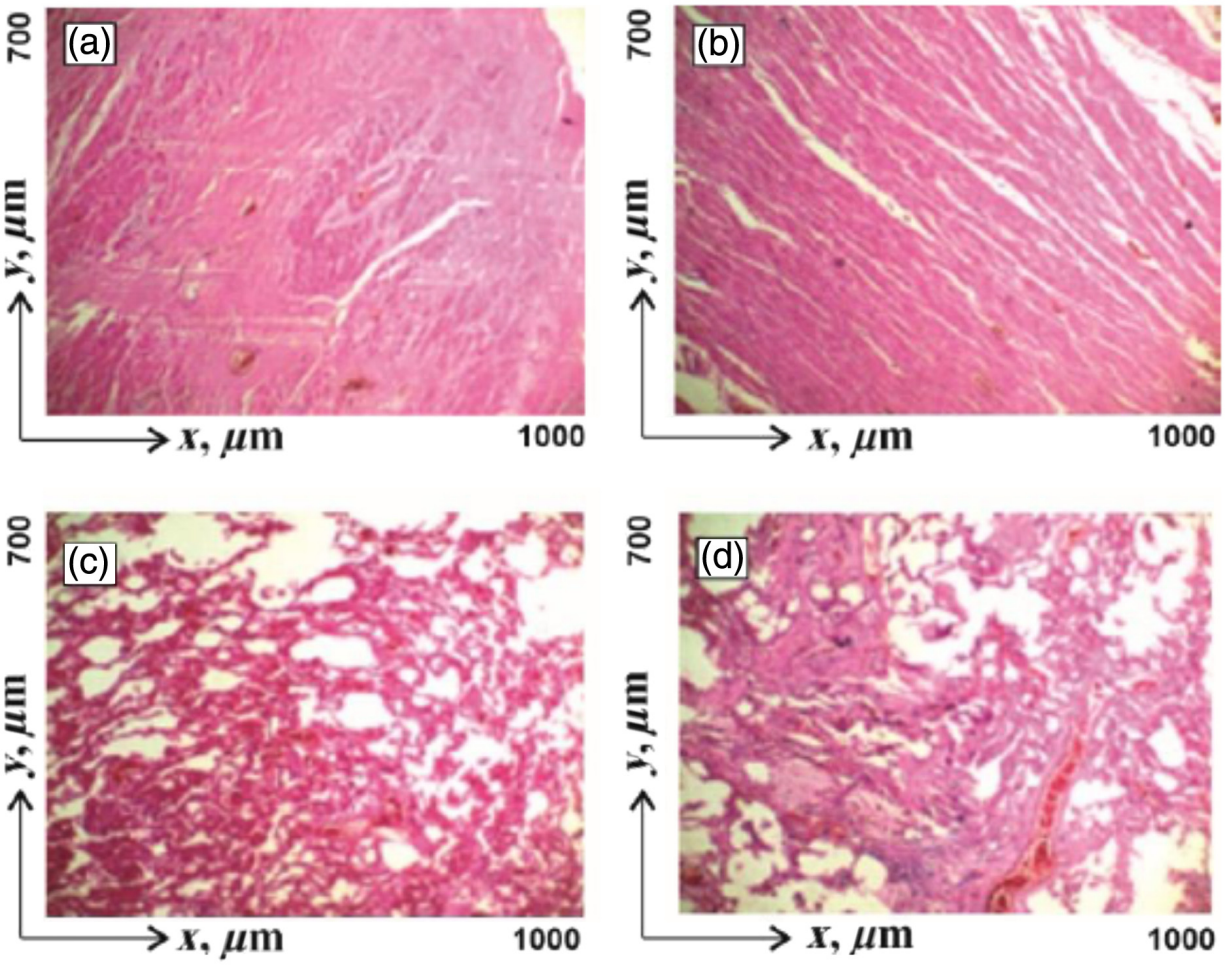
Microscopic images (×40) of histological preparations of myocardium and lung tissue. (a) CHD; (b) ACI; (c) BA; (d) PF. Explanations in the text.

Figure 2 shows microscopic images of all groups of histological preparations of the myocardium [CHD, Fig. 2(a), ACI, Fig. 2(b)] and lung tissue [BA, Fig. 2(c), PF, Fig. 2(d)].

Analysis of myocardial images from both groups reveals the presence of the fibrillar structure of myosin fibers [Figs. 2(a) and 2(b)]. A tissue sample with ACI is characterized by the presence of a spatially well-ordered fibrillar network with average geometric dimensions of 15 to 20  μm [Fig. 2(b)]. The myocardial fibrillar network with CHD is disordered and formed by thinner (10  μm) myosin fibrils [Fig. 2(a)].

The analysis of images of lung tissue with BA shows a weakly structured parenchymal morphological structure [Fig. 2(c)]. The image of the tissue with PF shows the presence of a formed fibrillar connective tissue component with an average fiber size of 5 to 10  μm [Fig. 2(d)].

This choice of objects is related to both the fundamental and applied components of our research.

#### 2.4.1 Fundamental component

From the physical point of view, the selected tissues have different optically anisotropic architectonics.

The myocardium is characterized by spatially structured linearly birefringent [ratio (2)] networks of myosin fibrils.^5^^,^^22^^,^^23^^,^^35^

The lung tissue is predominantly parenchymal with a slight linear birefringence of the collagen fibrillar networks of the connective tissue collagen component.^5^^,^^29^

Cases of CHD and ACI lead to various necrotic changes of optical anisotropy in myosin fibers and their spatially structured networks. CHD-myosin fibers thin out and the spatial order of fibrillar networks decreases [Fig. 2(a)]. As a result, the magnitude of the structural anisotropy decreases. ACI-myosin fibers are broken in some areas with constant special ordering of the music network [Fig. 2(b)]. Therefore, the birefringence level remains commensurate with the same anisotropy parameter of healthy tissue. BA does not significantly change the architectonics of collagen fibrillar networks of the connective tissue component [Fig. 2(c)]. PF is accompanied by a significant growth of collagen fibers and an increase in birefringence [Fig. 2(d)].

Therefore, a comparative physical analysis of the results obtained will allow us to determine the capabilities of our method in detecting changes in the optically anisotropic architectonics of biological tissue samples with different morphologies.

#### 2.4.2 Applied component

An important and not fully solved forensic task by light microscopy methods is to determine the natural (CHD) or violent (ACI) cause of myocardial death. The main problem for histological diagnosis is the presence of a high level of diffuse background in the images of histological sections of the myocardium.

The optical and geometric parameters of the diffuse histological section samples are presented in Table 1.

**Table 1:** Optical and geometric parameters of histological section tissues samples of both types.

Parameters	Myocardium	Lung’s tissue
Geometric thickness, h (μm)	50 to 60	50 to 60
Optical thickness, τ (μm)	0.18 to 0.22	0.19 to 0.23
Depolarization degree, Δ (%)	47 to 53	54 to 59

The extinction coefficient $\left(\tau ,{\mathrm{cm}}^{-1}\right)$ of the samples of biological tissues was measured according to the standard method of photometry of the attenuation^59^ of the intensity of the illuminating beam by the sample using an integral light-scattering sphere.^60^ The value of the integral degree of depolarization (Δ,%) of the biological tissues samples was measured in the scheme of a standard Mueller-matrix polarimeter.^5^^,^^22^^–^^25^^,^^50^^–^^52^

Histological sections were prepared using the conventional technique on a microtome with rapid freezing.^29^

### 2.5 Information Analysis

For the myocardium, our main applied task was to determine the possibility of detecting ACI cases at a high level of depolarized background. In this sense, the samples of histological sections of the myocardium of those who died from CHD formed control group 1. Accordingly, group 2 of myocardial samples of those who died from ACI was experimental.

The situation was different with lung tissue differentiation. The diagnostic purpose was to determine FP. Therefore, histological sections of lung tissue with BA formed the control group 3, and tissue samples with fibrosis formed the experimental group 4.

Information analysis of the results of polarization-interference phase scanning uses a number of operational characteristics of evidence-based medicine:^61^

• Sensitivity (Se) is the proportion of correct positive results (A) of the diagnostic method among all samples from experimental group 2, group 4 (N):
  Se=(A/N)100%. (36)
• Specificity (Sp) is the proportion of correct negative results (B) of the method among control group 1, group 3 (H):
  Sp=(B/H)100%. (37)
• Accuracy (Ac): proportion of corrPect test results (A+B) among all samples (N+H)
  Ac=[(A+B)/(N+H)]100%. (38)
  If (A+B)=(N+H), then Ac is called the balanced accuracy.

In our work, the following scale for evaluating the diagnostic accuracy is used (Table 2).

**Table 2:** Threshold levels of balanced accuracy.

Diagnostic accuracy assessment	Accuracy, Ac(%)
Unsatisfactory	≤80
Satisfactory	81 to 85
Good	86 to 90
Very good	91 to 95
Excellent	>95

## 3 Results and Discussion

By the method of phase scanning [ratios (27)–(29)], we have determined the phase plane of a single scattering [ratio (31)]. All further discussion refers to the experimental results obtained in this plane $\delta_{t}^{⋆}\leq \pi /8$.

### 3.1 Wavelet Differentiation of Azimuth Polarization Map of the Myocardium

**Fig. 3:**
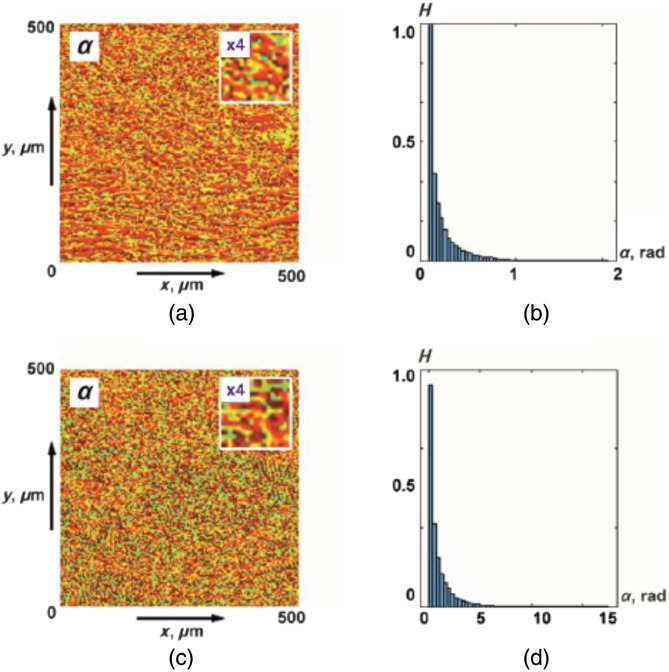
(a), (b) Coordinate and (c), (d) probabilistic distributions of random values of the azimuth of polarization of object fields of myocardial samples of deceased as a result of (a), (c) ACI and (b), (d) CHD.

Figure 3 shows the maps and histograms of distributions of random values of the azimuth of polarization of object fields of the myocardium from group 1 and group 2.

As an initial reference for the azimuths of polarization ($\alpha^{j=1}(\delta_{t}^{⋆}=\pi /8)$), we used the ratio $⊗↔\alpha^{j=1}=0$.

The analysis of the obtained data revealed the presence of a coordinate-heterogeneous [Figs. 3(a) and 3(c)] and statistically distributed [Figs. 3(b) and 3(d)] structure of polarization azimuth maps for object fields of myocardial samples from both groups.

Topologically, azimuth polarization maps are formed by local domains with different geometric dimensions [Figs. 3(a) and 3(c)]. The distribution of the scales of such polarization domains [ratios (2) to (7)] is individual for the object fields of myocardial samples from different groups. This can be explained by the maximum correlation in the phase plane of a single scattering of the scales of optically anisotropic fibrillar structures of the myocardium and polarization domains. At the same time, the histograms of the distributions [Figs. 3(b) and 3(d)] are quite similar in localization of extremes and ranges of variation of random values of the azimuth of polarization. In other words, statistical distributions of polarization parameters do not provide information about the large-scale (topological) structure of necrotic changes in myocardial architectonics.

**Fig. 4:**
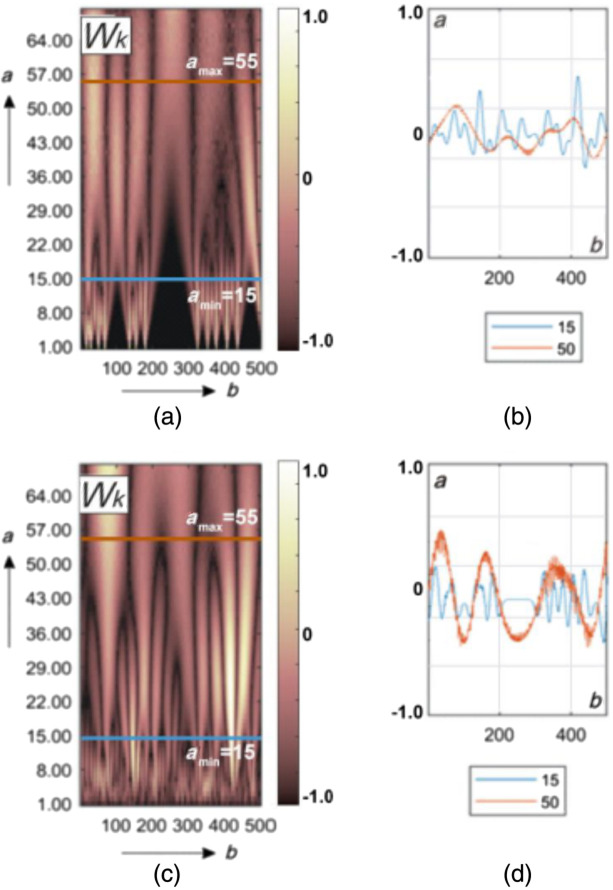
Maps (left column) and multi-scale linear cross-sections (right column) of the polarization azimuth $\alpha (\delta_{t}^{⋆}=\pi /8,m,n)$ wavelet coefficients $W_{a,b}$ of myocardium histological sections of those who died from CHD (top row bottom row) and ACI (bottom row). Two-dimensional array of values of the amplitudes of the wavelet coefficients (a), (c) and linear distributions of the amplitude of the wavelet coefficients for two scales of the MHAT function (b), (d).

Figure 4 shows the results of wavelet analysis of the polarization azimuth $\alpha (\delta_{t}^{⋆}\leq \pi /8,m,n)$ maps of the myocardium histological sections microscopic images.

This drawing consists of two parts.

Figures 4(a) and 4(c) show a two-dimensional array of values of the amplitudes of the wavelet coefficients. It is formed as a result of the algorithmic [ratios (24) to (26)] line-by-line scanning of MHAT distributions by a variable-scale function $\Psi (\frac{x-b}{a})$.

Figures 4(b) and 4(d) show an example of linear distributions of the amplitude of the wavelet coefficients for two scales of the MHAT function.

It can be seen from the data obtained that the 2D amplitude distributions of the wavelet coefficients represent $W_{a,b}$ a fluctuating surface [Figs. 4(a) and 4(c)]. The amplitudes and the oscillation period are distributed differently for different scales a of the salt-like MHAT function $\Psi (\frac{x-b}{a})$. As the scale increases (a↑), the oscillation period of the linear distributions of the wavelet coefficients $W_{b}$ increases. The amplitude modulation depth of the wavelet coefficients $W_{b}$ is individual for different scales [Figs. 4(b) and 4(d)].

The revealed patterns can be explained by the following considerations. The magnitude of the amplitude of the wavelet coefficient at each scanning point is determined by the degree of mutual correlation of the geometric parameters of the polarization domain [Figs. 3(a) and 3(c)] and the scale of the “window” of the MHAT function $\Psi (\frac{x-b}{a})$. The greater the cross-correlation, the greater the amplitude of the wavelet coefficient.

On small scales of polarization maps, such wavelet correlations can be traced for fine-structured elements of myocardial fibrillar architectonics. For large scales, variations in the amplitude of the wavelet coefficients are associated with the degree of ordering of large-scale fibers of the fibrillar network.

In the case of death as a result of ACI, the fibrillar myosin network is more ordered and broadly structured in comparison with the architectonics of the myocardium of those who died from CHD [Figs. 2(a) and 2(b)]. Therefore, as a result of the wavelet transform of the polarization maps of the myocardial object field for the sample from group 2, more amplitude values and a greater depth of modulation of their changes are formed [Figs. 4(a), 4(b) and 4(c), 4(d)].

Quantitatively, we determined the differences between the polarization maps of the myocardial object fields in two ways.

The first is a direct calculation method [ratio (30)] statistical moments that characterize the distributions (Table 3).

**Table 3:** Statistical moments of the polarization azimuth maps $\alpha (\delta_{t}^{⋆}=\pi /8,m,n)$.

$Z_{1,2,3,4}$	CHD	ACI	*Ac* (%)
$Z_{1}$	0.07 ± 0.005	0.09 ± 0.006	70.8
$Z_{2}$	0.04 ± 0.003	0.05 ± 0.003	75
$Z_{3}$	1.73 ± 0.095	1.29 ± 0.067	79.2
$Z_{4}$	2.39 ± 0.13	2.11 ± 0.12	79.2

From the analysis of the data obtained, it can be seen that the maximum accuracy of the differential diagnosis of CHD and ACI practically achieved only a satisfactory level $\mathrm{Ac}(Z_{3,4}=79.2\%)$.

**Fig. 5:**
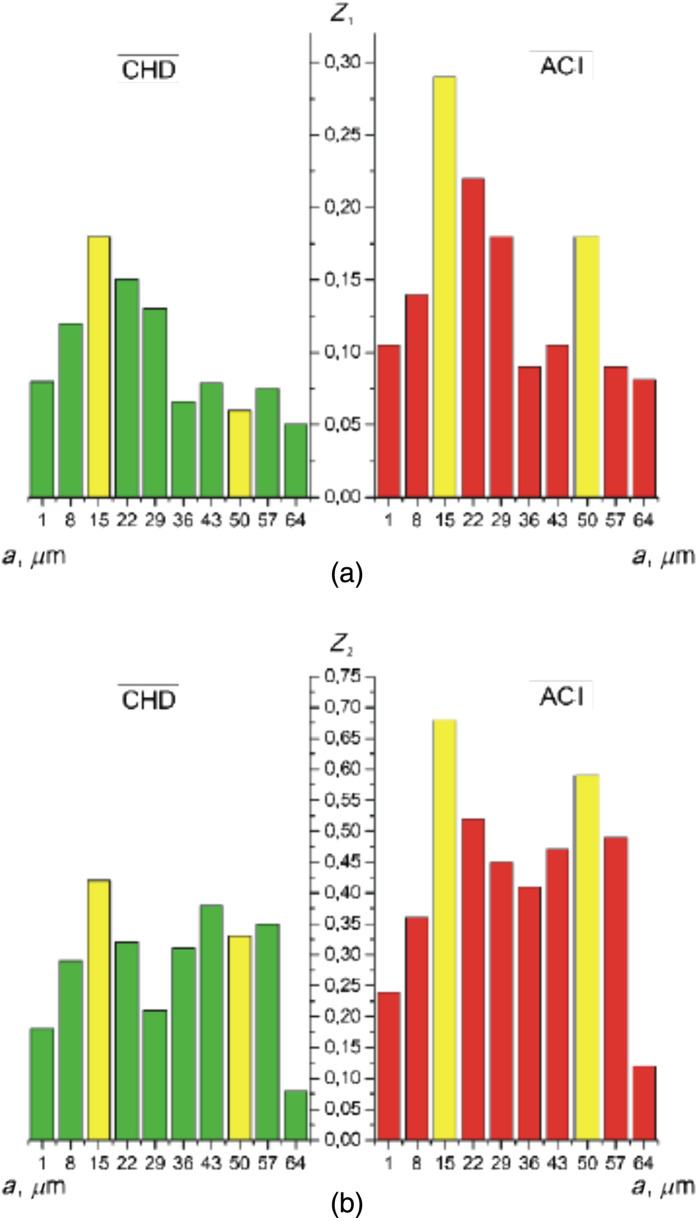
Scale dependences of (a) the mean $Z_{1}$ and (b) the variance $Z_{2}$ of the wavelet coefficients $W_{a,b}$ of the $\alpha (\delta_{t}^{⋆}=\pi /8,m,n)$. Explanation in the text.

The second is a method for estimating fluctuations in the amplitude of the wavelet coefficients by calculating [ratios (31) and (32)] statistical parameters [mean $Z_{1}(W_{a,b})$ and variation $Z_{2}(W_{a,b})$] for all scales of the MHAT function (Fig. 5). Here, based on the obtained statistical analysis data, the scales for which the differences were determined. The differences between $Z_{1}(W_{a,b})$ and $Z_{2}(W_{a,b})$ were maximal (Table 4). From the obtained data, it was revealed individual modulation of the amplitude values $W_{b}(a_{\min}=15)$ and $W_{b}(a_{\max}=55)$.

**Table 4:** Mean $Z_{1}$ and variance $Z_{2}$ of the wavelet coefficients $W_{a,b}$ of the $\alpha (\delta_{t}^{⋆}=\pi /8,m,n)$.

$a_{\min}=15$			
$Z_{i}$	CHD	ACI	*Ac* (%)
$Z_{1}$	0.18 ± 0.01	0.29 ± 0.016	91.7
$Z_{2}$	0.46 ± 0.025	0.68 ± 0.036	95.8
$a_{\max}=50$			
$Z_{i}$	CHD	ACI	*Ac* (%)
$Z_{1}$	0.09 ± 0.005	0.12 ± 0.007	87.5
$Z_{2}$	0.43 ± 0.023	0.55 ± 0.029	87.5

For the case of CHD, the maximum amplitude and variations in the wavelet value of the Wa,b coefficients occur on large scales of the wavelet function scanning in the phase plane $\delta_{t}^{⋆}=\pi /8$ of the $\alpha (\delta_{t}^{⋆}=\pi /8,m,n)$ map.

For the case of ACI, the maximum amplitude and variations in the wavelet value of the $W_{a,b}$ coefficients occur on small scales of the wavelet function scanning in the phase plane $\delta_{t}^{⋆}=\pi /8$ of the $\alpha (\delta_{t}^{⋆}=\pi /8,m,n)$ map.

Analysis of statistical estimation of fluctuations in the amplitudes of multiscale wavelet coefficients $W_{a,b}$ distributions of the polarization azimuth $\alpha (\delta_{t}^{⋆}=\pi /8,m,n)$ revealed next ratios $Z_{1,2}(\mathrm{ACI},W_{b}(a_{\min}=15))\ll Z_{1,2}(\mathrm{CHD},W_{b}(a_{\min}=15))$ and $Z_{1,2}(\mathrm{ACI},W_{b}(a_{\max}=55))<Z_{1,2}(\mathrm{CHD},W_{b}(a_{\max}=55)$. As a result, the following levels of accuracy of differential diagnosis of cases were determined CHD and ACI. For small scales, $a_{\min}=15$- very good $\mathrm{Ac}(Z_{1})=91.7\%$ and excellent $\mathrm{Ac}(Z_{2})=95.8\%$ are equal. For large scales, $a_{\max}=50$ is a good level of $\mathrm{Ac}(Z_{1,2})=87.5\%$.

### 3.2 Wavelet Differentiation of the Ellipticity Polarization Maps of Myocardium

**Fig. 6:**
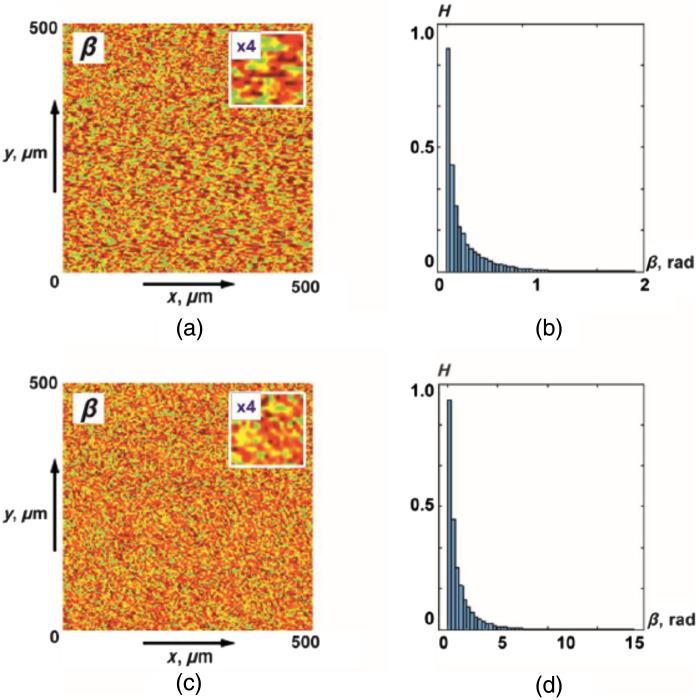
(a), (c) Coordinate and (b), (d) probabilistic distributions of random values of the magnitude of the ellipticity of polarization of object fields of myocardial samples that died as a result of (a), (b) CHD and (3), (4) ACI.

Figure 6 shows maps and histograms of distributions of random values of the magnitude of ellipticity of polarization of object fields of histological sections of the myocardium from group 1 and group 2.

As an initial reference for the ellipticity of polarization ($\beta^{j=1}(\delta_{t}^{⋆}=\pi /8)$), we used the ratio $⊗↔\beta^{j=1}=0$.

As for the polarization azimuth maps (Fig. 3), the coordinate distributions of ellipticity turned out to be coordinate-inhomogeneous [Figs. 6(a) and 6(c)] and statistically distributed [Figs. 6(b) and 6(d)].

It can be seen that the set of polarization domains of ellipticity maps [ratios (7), (8), (11), (12)] is individual for the object fields of myocardial samples from different groups. At the same time, statistical distributions of random values of the magnitude of the ellipticity of polarization do not carry information about the large-scale (topological) structure of necrotic changes in the architectonics of birefringent fibrillar networks of the myocardium.

Table 5 presents the results of a statistical analysis of the distributions of the ellipticity of polarization of myocardial samples from the groups CHD and ACI.

**Table 5:** Statistical moments of polarization ellipticity maps of the $\beta (\delta_{t}^{⋆}=\pi /8,m,n)$.

$Z_{1,2,3,4}$	CHD	ACI	*Ac* (%)
$Z_{1}$	0.11 ± 0.007	0.13 ± 0.008	70.8
$Z_{2}$	0.07 ± 0.005	0.09 ± 0.006	75
$Z_{3}$	1.21 ± 0.069	0.93 ± 0.057	79.2
$Z_{4}$	1.87 ± 0.099	1.36 ± 0.079	83.3

It was found that the maximum accuracy of differential diagnostics of CHD and ACI exceed a satisfactory level $\mathrm{Ac}(Z_{4}=83.3\%)$.

**Fig. 7:**
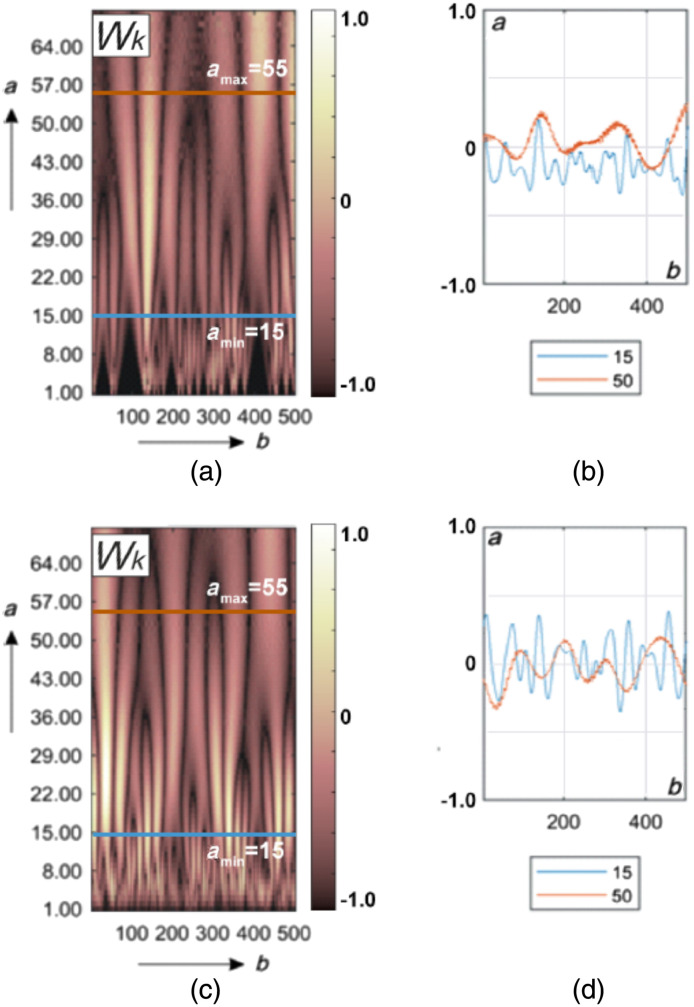
Maps (left column) and multi-scale linear cross-sections (right column) of the polarization ellipticity $\beta (\delta_{t}^{⋆}=\pi /8,m,n)$ wavelet coefficients $W_{a,b}$ of myocardium histological sections of those who died from CHD (top row) and ACI (bottom row). Two-dimensional array of values of the amplitudes of the wavelet coefficients (a), (c) and linear distributions of the amplitude of the wavelet coefficients for two scales of the MHAT function (b), (d).

Figure 7 shows the results of the application wavelet analysis technique W(a,b) for maps of the ellipticity $\beta (\delta_{t}^{⋆}=\pi /8,m,n)$.

We obtained fluctuating surfaces of the amplitudes of the wavelet coefficients $W_{a,b}$ [Figs. 7(a) and 6(c)]. The amplitudes and the oscillation period of the linear distributions of the wavelet coefficients increase as the scale a of the MHAT function $\Psi (\frac{x-b}{a})$ increases. The depth of modulation of the amplitude of the wavelet coefficients of the polarization ellipticity maps (Fig. 6) is individual for different scales [Figs. 7(b) and 7(d)].

At all scales of polarization maps of ellipticity, the wavelet correlations are determined by the level of structural anisotropy or birefringence.

The value of this optical anisotropy parameter is related to the degree of spatial ordering of the fibers of the fibrillar network.

Therefore, in case of death, as a result of ACI, more amplitude values are formed and the depth of modulation of their changes is greater [Figs. 4(a), 4(b) and 4(c), 4(d)].

**Fig. 8:**
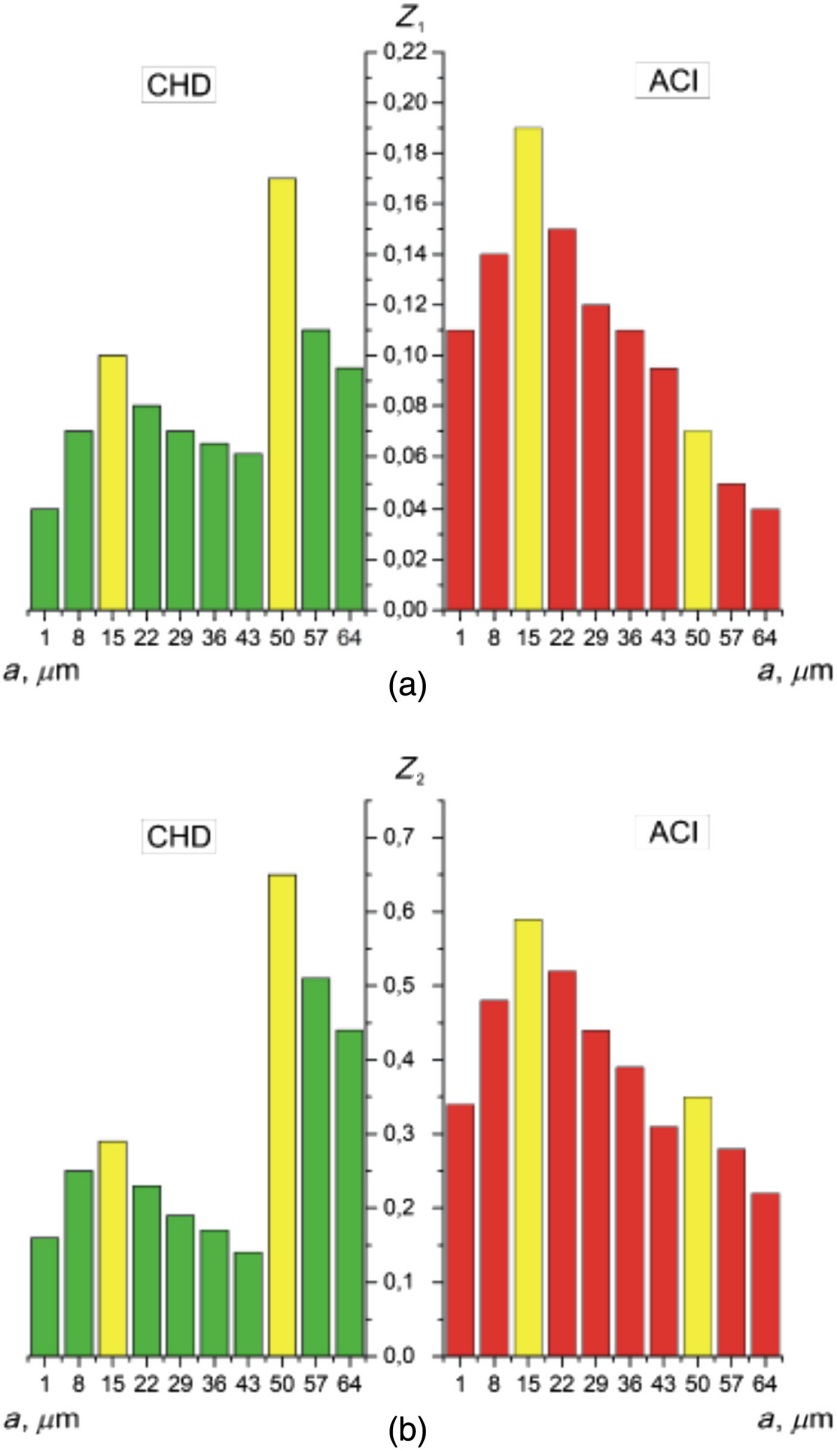
Scale dependences of (a) the mean $Z_{1}$ and (b) the variance $Z_{2}$ of the wavelet coefficients $W_{a,b}$ of the $\beta (\delta_{t}^{⋆}=\pi /8,m,n)$. Explanation in the text.

We quantified such amplitude fluctuations of the wavelet coefficients $W_{a,b}$ by calculating statistical parameters [$Z_{1}(W_{a,b})$ and $Z_{2}(W_{a,b})$] for all scales a of the MHAT function $\Psi (\frac{x-b}{a})$ (Fig. 8, Table 4).

High levels of differential diagnosis accuracy have been established for small scales $a_{\min}=15$ is an excellent level of $\mathrm{Ac}(Z_{1})=95.8\%$ and $\mathrm{Ac}(Z_{2})=100\%$. For large scales $a_{\max}=50$ is a very good level of $\mathrm{Ac}(Z_{1,2})=91.7\%$ (Table 6).

**Table 6:** Mean $Z_{1}$ and variance $Z_{2}$ of wavelet coefficients of polarization ellipticity maps $\beta (\delta_{t}^{⋆}=\pi /8,m,n)$.

$a_{\min}=15$			
$Z_{i}$	CHD	ACI	*Ac* (%)
$Z_{1}$	0.11 ± 0.006	0.18 ± 0.01	95.8
$Z_{2}$	0.31 ± 0.016	0.59 ± 0.031	100
$a_{\max}=50$			
$Z_{i}$	CHD	ACI	*Ac* (%)
$Z_{1}$	0.08 ± 0.005	0.14 ± 0.008	91.7
$Z_{2}$	0.39 ± 0.021	0.62 ± 0.033	91.7

### 3.3 Wavelet Differentiation of the Polarization Azimuth Maps of Lung Tissue

**Fig. 9:**
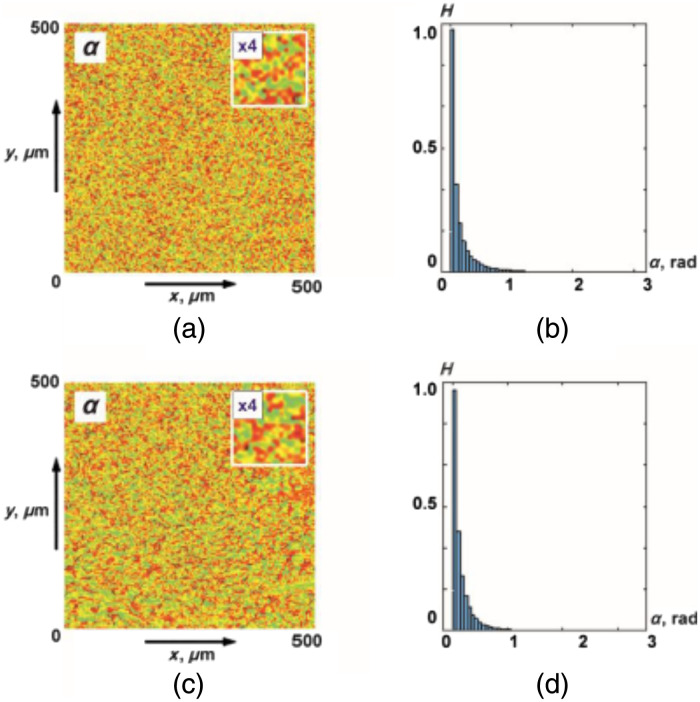
(a), (c) Coordinate and (b), (d) probabilistic distributions of random values of the azimuth of polarization of object fields of lung tissue samples with (a), (b) BA and (c), (d) PF.

Figure 9 shows maps and histograms of distributions of random values of the azimuth of polarization of object fields of histological sections of parenchymal lung tissue from group 3 and group 4.

The obtained data show the presence of a coordinate-inhomogeneous [Figs. 9(a) and 9(c)] and statistically distributed [Figs. 9(b) and 9(d)] structure of polarization azimuth maps for object fields of lung tissue samples from both groups.

As in the case of myocardial object fields (Figs. 3 and 6), azimuth polarization maps are formed by local domains with different geometric dimensions [Figs. 9(a) and 9(c)]. The histograms of the distributions [Figs. 9(b) and 9(d)] are quite similar in localization of extremes and ranges of variation of random values of the azimuth of polarization. As a result, the differences between the values of the statistical moments of the first to fourth orders $Z_{1;2;3;4}$ are insignificant and do not exceed 15% to 25% (Table 7).

**Table 7:** Statistical moments of the polarization azimuth maps of the $\alpha (\delta_{t}^{⋆}=\pi /8,m,n)$.

$Z_{1;2;3;4}$	BA	PF	*Ac* (%)
$Z_{1}$	0.09 ± 0.01	0.11 ± 0.016	66.7
$Z_{2}$	0.07 ± 0.025	0.08 ± 0.036	63.3
$Z_{3}$	1.12 ±0.005	0.94 ± 0.007	73.3
$Z_{4}$	1.71 ± 0.023	1.49 ± 0.029	76.7

From the analysis of the data obtained, it can be seen that the maximum accuracy of the differential diagnosis of CHD and ACI does not reach a satisfactory level $\mathrm{Ac}(Z_{1,2,3,4}<80\%)$.

**Fig. 10:**
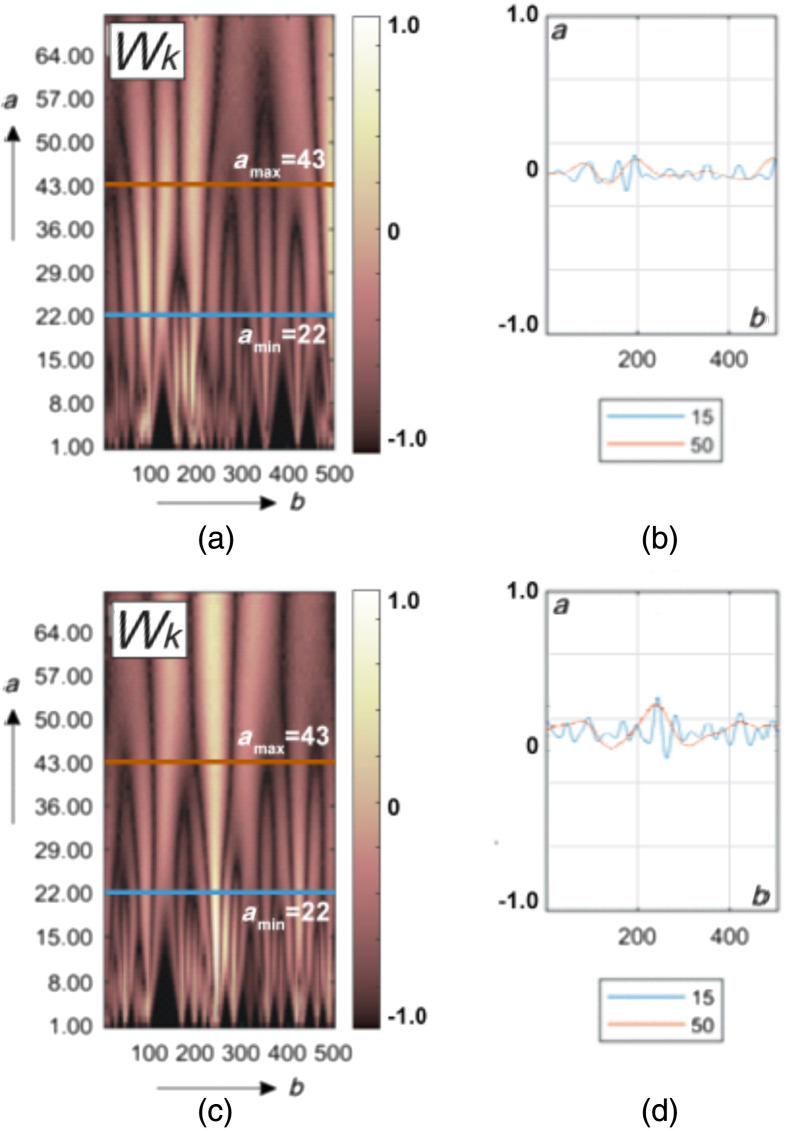
Maps (left column) and multi-scale linear cross-sections (right column) of the polarization azimuth $\alpha (\delta_{t}^{⋆}=\pi /8,m,n)$ wavelet coefficients $W_{a,b}$ of lung tissue histological sections those who died from BA (top row) and PF (bottom row). Explanation in the text.

Figure 10 presents the results of wavelet analysis of polarization maps $\alpha (\delta_{t}^{⋆}=\pi /8,m,n)$ for BA and PF cases.

The presence of individual fluctuations in the amplitude of the wavelet coefficients at all scales of the salt-like MHAT function was found [Figs. 10(a) and 10(c)].

As in the case of wavelet decomposition of polarization maps of myocardial samples, the magnitude of the amplitude at each scanning point is determined by the degree of mutual correlation of the geometric parameters of the polarization domain [Figs. 9(a) and 9(c)] and the scale of the “window” of the MHAT function.

In the case of BA, the optical birefringence of the pulmonary parenchyma is insignificant [Fig. 2(c)]. For the PF situation, the level of structural anisotropy increases due to the proliferation of connective tissue [Fig. 2(d)].

Therefore, as a result of the wavelet transform of the polarization maps of the sample from group 3, small amplitude values with a small modulation depth are formed in comparison with the data for the sample from group 4 [Figs. 10(a), (b) and (c), (d)].

**Fig. 11:**
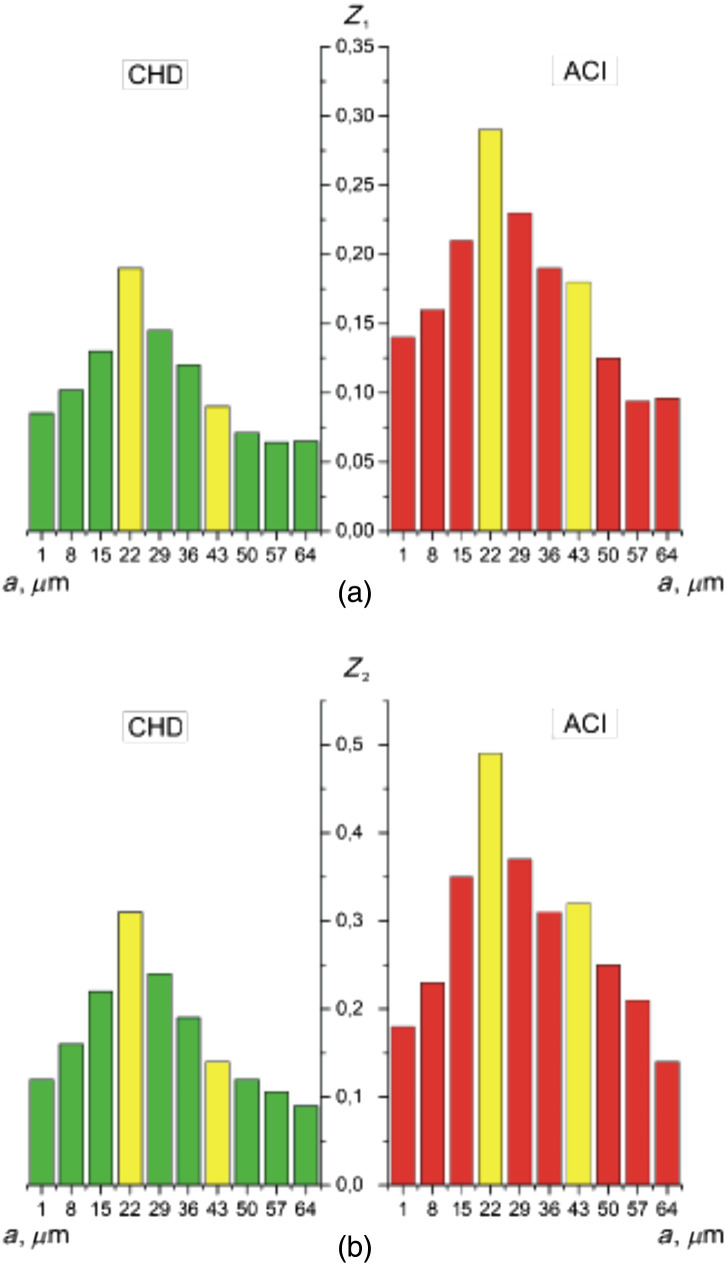
Scale dependences of (a) the mean $Z_{1}$ and (b) the variance $Z_{2}$ of the wavelet coefficients distributions for polarization azimuth maps $\alpha (\delta_{t}^{⋆}=\pi /8,m,n)$. Two-dimensional array of values of the amplitudes of the wavelet coefficients (a), (c) and linear distributions of the amplitude of the wavelet coefficients for two scales of the MHAT function (b), (d).

We quantified such fluctuations in the amplitude of the wavelet coefficients $W_{a,b}$ by calculating statistical parameters ($Z_{1}(W_{a,b})$) and variation $Z_{2}(W_{a,b})$ for all scales a of the MHAT function $\Psi (\frac{x-b}{a})$ (Fig. 11). The scales proved to be diagnostically optimal $a_{\min}=22$ and $a_{\max}=43$ (Table 8).

**Table 8:** Mean $Z_{1}$ and the variance $Z_{2}$ of the wavelet coefficients distributions for polarization azimuth maps $\alpha (\delta_{t}^{⋆}=\pi /8,m,n)$.

$a_{\min}=22$			
$Z_{i}$	BA	PF	Ac (%)
$Z_{1}$	0.19 ± 0.01	0.29 ± 0.016	90
$Z_{2}$	0.31 ± 0.017	0.49 ± 0.027	93.3
$a_{\max}=43$			
$Z_{i}$	BA	PF	Ac(%)
$Z_{1}$	0.09 ± 0.005	0.16 ± 0.09	93.3
$Z_{2}$	0.14 ± 0.08	0.32 ± 0.018	96.7

High levels of differential diagnosis accuracy have been established. For small scales, $a_{\min}=22$ is a good and very good level of $\mathrm{Ac}(Z_{1})=90\%$ and $\mathrm{Ac}(Z_{2})=93.3\%$. For large scales, $a_{\max}=43$ is a very good $Ac(Z_{1})=93.3\%$ and excellent $Ac(Z_{2})=96.7\%$ level.

The obtained results can be physically related to changes in all scales of geometric dimensions of parenchymal connective tissue birefringence fibrils in the lung parenchyma volume in the case of fibrosis. Therefore, at all scales, there is a maximum modulation of the polarization azimuth $\alpha (\delta_{t}^{⋆}=\pi /8,m,n)$ and, accordingly, variations in the amplitudes of the wavelet coefficients $W_{a,b}$. For BA, the modulation of the wavelet coefficients is minimal.

### 3.4 Wavelet Differentiation of Ellipticity Polarization Maps of Lung Tissue

**Fig. 12:**
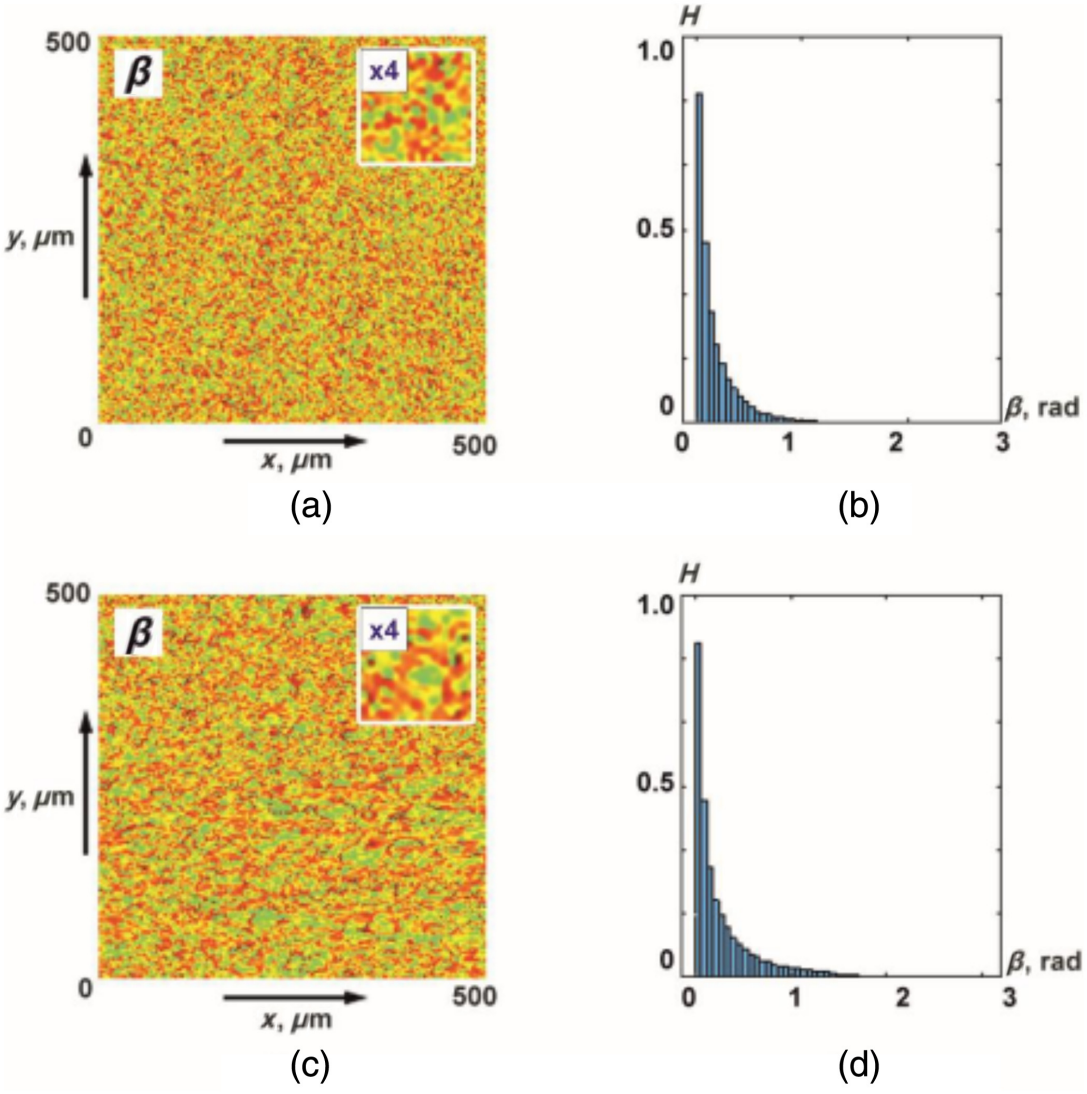
(a), (c) Coordinate and (b), (d) probabilistic distributions of random values of the magnitude of the ellipticity of polarization of object fields of lung tissue samples of deceased as a result of (a), (b) BA and (c), (d) PF.

Figure 12 shows maps and histograms of distributions of random values of the magnitude of ellipticity of polarization of object fields of histological sections of the lung tissue from group 3 and group 4.

As for the polarization azimuth maps (Fig. 9), the coordinate distributions of the ellipticity of the object field of lung tissue samples turned out to be coordinate-inhomogeneous [Figs. 12(a) and 12(c)]. The histograms of the distributions of random values of the ellipticity of the object fields of the samples from group 3 and group 4 are quite similar [Figs. 12(b) and 12(d)]. As a result, the differences between the values of statistical moments of the first to fourth $Z_{1,2,3,4}$ orders are 25% to 35% (Table 9).

**Table 9:** Statistical moments $Z_{1,2,3,4}$ of polarization ellipticity maps of the $\beta (\delta_{t}^{⋆}=\pi /8,m,n)$.

$Z_{1,2,3,4}$	BA	PF	*Ac* (%)
$Z_{1}$	0.05 ± 0.003	0.08 ± 0.006	80
$Z_{2}$	0.03 ± 0.002	0.05 ± 0.003	80
$Z_{3}$	1.44 ± 0.075	1.03 ± 0.057	83.3
$Z_{4}$	2.06 ± 0.11	1.61 ± 0.089	83.3

It can be seen that the maximum accuracy of the differential diagnosis of BA and PF corresponds to a satisfactory level $\mathrm{Ac}(Z_{1,2,3,4})=80\%$ to 83.3%. At the same time, the differences in the topological structure of the polarization ellipticity maps are more pronounced for the fields of histological sections of lung tissue from group 3 and group 4.

**Fig. 13:**
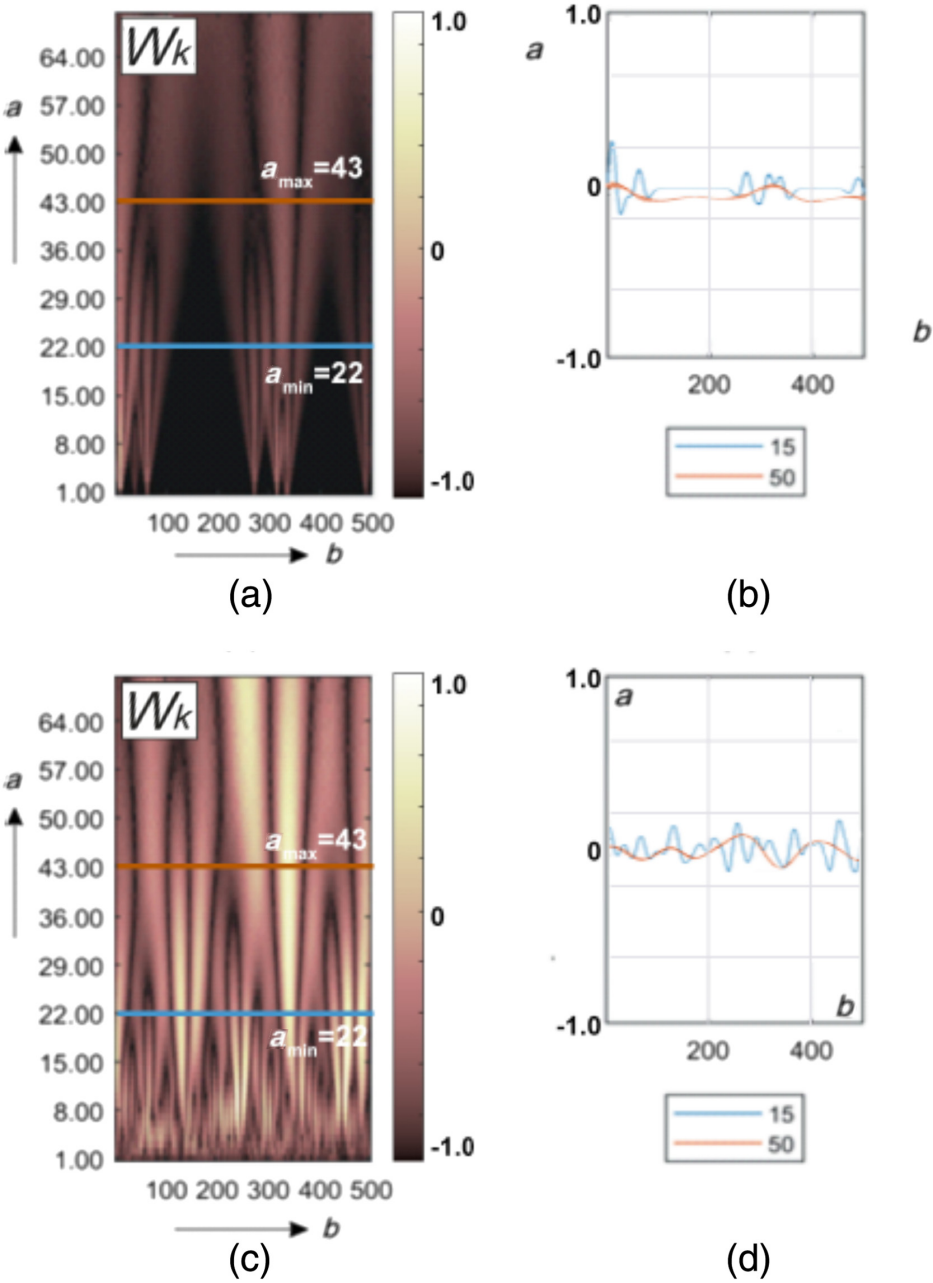
Maps $\beta (\delta_{t}^{⋆}=\pi /8,m,n)$ (left column) and multi-scale linear cross-sections (right column) of the polarization ellipticity wavelet coefficients $W_{a,b}$ of lung tissue histological sections those who died from BA (top row) and PF (bottom row). Explanation in the text.

Figure 13 shows wavelet maps W(a,b) of $\beta (\delta_{t}^{⋆}=\pi /8,m,n)$ for BA and PF cases.

**Fig. 14:**
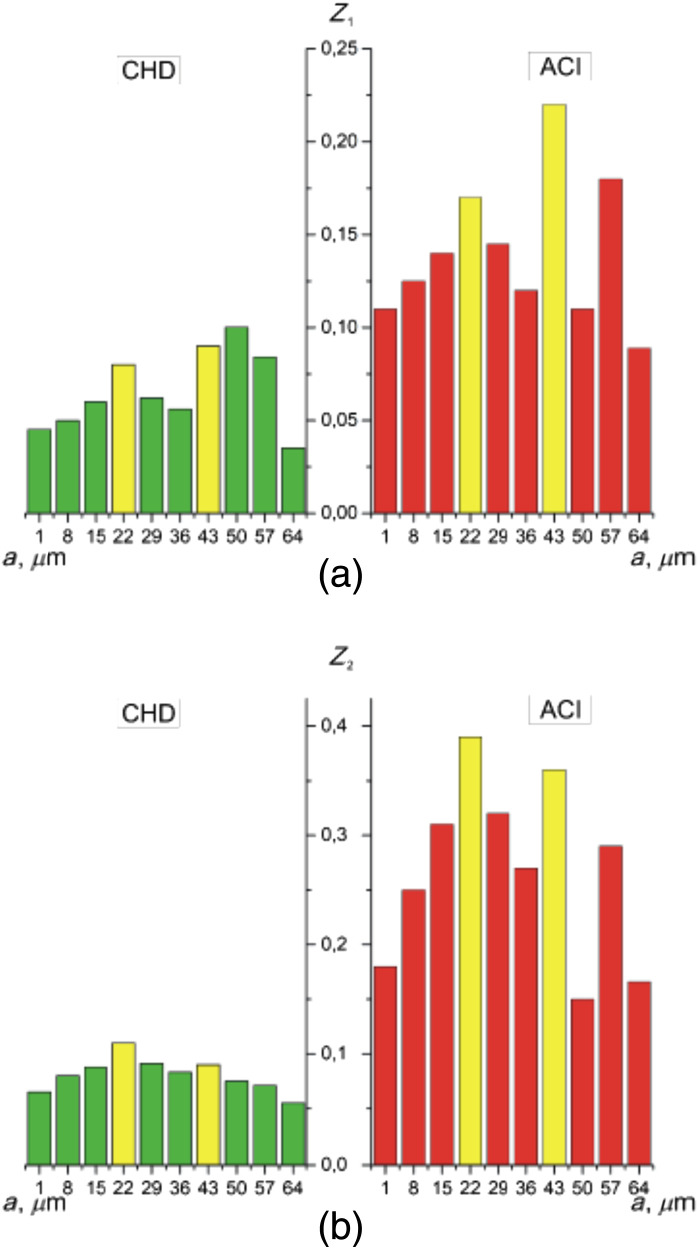
Scale dependences of (a) the mean $Z_{1}$ and (b) the variance $Z_{2}$ of wavelet coefficients distributions for $\beta (\delta_{t}^{⋆}=\pi /8,m,n)$. Explanation in the text.

As can be seen (Table 10), the value of the mean and variance of linear dependencies of the wavelet coefficients of ellipticity of maps of lung tissue samples from group 4 (PF) is three to four times greater than similar statistical parameters for the case of BA. Thus, high levels of accuracy in the differential diagnosis of cases of BA and PF have been established. An excellent accuracy level of 96.7% to 100% was obtained on all a scales of MHAT function $\Psi (\frac{x-b}{a})$ (Fig. 14).

**Table 10:** Mean $Z_{1}$ and the variance $Z_{2}$ of the wavelet coefficients distributions for polarization azimuth maps $\beta (\delta_{t}^{⋆}=\pi /8,m,n)$.

$a_{\min}=22$			
$Z_{i}$	BA	PF	*Ac* (%)
$Z_{1}$	0.13 ± 0.008	0.17 ± 0.009	96.7
$Z_{2}$	0.36 ± 0.021	0.64 ± 0.038	100
$a_{\max}=43$			
$Z_{i}$	BA	PF	*Ac* (%)
$Z_{1}$	0.078 ± 0.042	0.135 ± 0.007	100
$Z_{2}$	0.19 ± 0.011	0.56 ± 0.031	100

## 4 Conclusions

1. The polarization-interference method of mapping and phase selection of diffuse layers of biological tissues with different morphological structure scattered with different multiplicities of polarization-inhomogeneous components of the object field is analytically substantiated and experimentally tested.
2. By the method of digital holographic reconstruction with phase scanning of complex amplitude distributions, polarization maps of a single scattered component in the object field of diffuse depolarizing histological sections of fibrillar myocardium and parenchymal lung tissue with the following types of pathology were algorithmically obtained:
  • myocardium: CHD – ACI;
  • lung tissue: asthma (BA) – fibrosis (PF).
3. Using the scanning soliton-shaped MHAT function, a scale-selective wavelet decomposition of a series of polarization maps of azimuth and ellipticity of polarization is implemented. The geometric scales of the structural elements of the polarization maps of a single scattered component in diffuse fields have been determined and physically justified to differentiate pathological changes in the optically anisotropic architectonics of myocardial and lung tissue samples.
4. For the obtained scales, within the framework of statistical analysis of linear distributions of the magnitude of the amplitudes of the wavelet coefficients, markers of differential diagnosis are determined CHD-ACI and BA-PF.
5. Diagnostic relationships between first- and fourth orders statistical moments, which characterize the wavelets coefficients distributions of azimuth and ellipticity maps, and diagnostic levels of various pathological conditions differentiation accuracy are revealed. For cases “CHD-ACI” installed very good $\mathrm{Ac}(Z_{1,2};a_{\min}=15)=91.7\%$ excellent level of $\mathrm{Ac}(Z_{1})=95.8\%$ and $\mathrm{Ac}(Z_{2};a_{\max}=50)=100\%$. For cases “BA–PF” installed very good $\mathrm{Ac}(Z_{1};a_{\min}=22)=92.3\%$ and excellent $\mathrm{Ac}(Z_{2};a_{\min}=22;a_{\max}=43)=100\%$ levels.

## Data Availability

The resulting data (figures) are uploaded to Figshare.^62^
